# Behcet’s disease involved the root of aorta in the treatment with modified Bentall procedure: a case report

**DOI:** 10.1186/s13019-020-1070-0

**Published:** 2020-01-29

**Authors:** Zeyi Cheng, Zhefeng Kang, Yupeng Ji, Yingqiang Guo

**Affiliations:** Department of Cardiovascular Surgery, West China Hospital, Sichuan University, Chengdu, Sichuan 610041 People’s Republic of China

**Keywords:** Behcet’s disease, Aortic valve regurgitation, Aortic sinus pseudoaneurysm, Bentall procedure

## Abstract

**Background:**

Behcet’s disease (BD) is a multisystemic vasculitis of unknown etiology, the incidence of cardiovascular system involvement is rare, about1–5% (Sakane et al., N Engl J Med 341:1284–91, 1999). BD combined with aortic pseudoaneurysm and aortic valve regurgitation is usually need surgical treatment, but there is controversy about which surgical method to choose.

**Case presentation:**

We report a case of BD combined with severe aortic valve regurgitation and two giant pseudoaneurysms of the aortic sinus. The patient underwent modified Bentall procedure (MBP) and use oral immunosuppressive as well as corticosteroid strictly, after 8 months follow-up, the patient recovered well.

**Conclusion:**

For patient with aortic valve regurgitation and ascend aortic pseudoaneurysm caused by BD, we recommend modified Bentall procedure when rheumatism in a stable period. Corticosteroids and immunosuppressive drugs should be used before and after surgery.

## Background

BD is a systematic chronic vasculitis that involves multiple systems, but the mechanism of BD still unclear. The main clinical manifestations include oral ulcers, genital ulcers, ophthalmia, skin lesions, Vascular, gastrointestinal, neurological systems may be also involved. BD combined with aortic pseudoaneurysm and aortic valve regurgitation is rare, and in majority of cases usually died for vascular complications. we report a case of BD combined with aortic valve regurgitation and two giant pseudoaneurysms of the aortic sinus, the patient was successfully treated by modified Bentall procedure.

### Case presentation

**Fig. 1:**
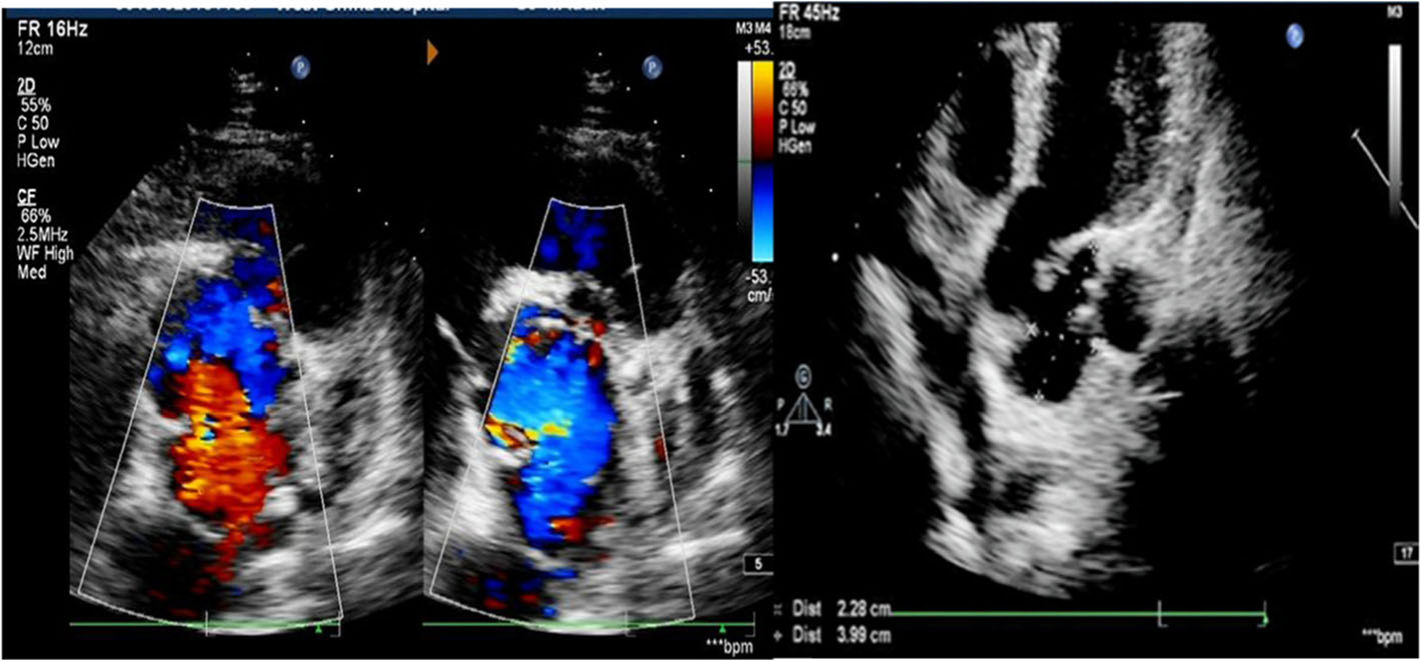
TTE demonstrated aortic valve regurgitation, aortic sinus pseudoaneurysms

**Fig. 2:**
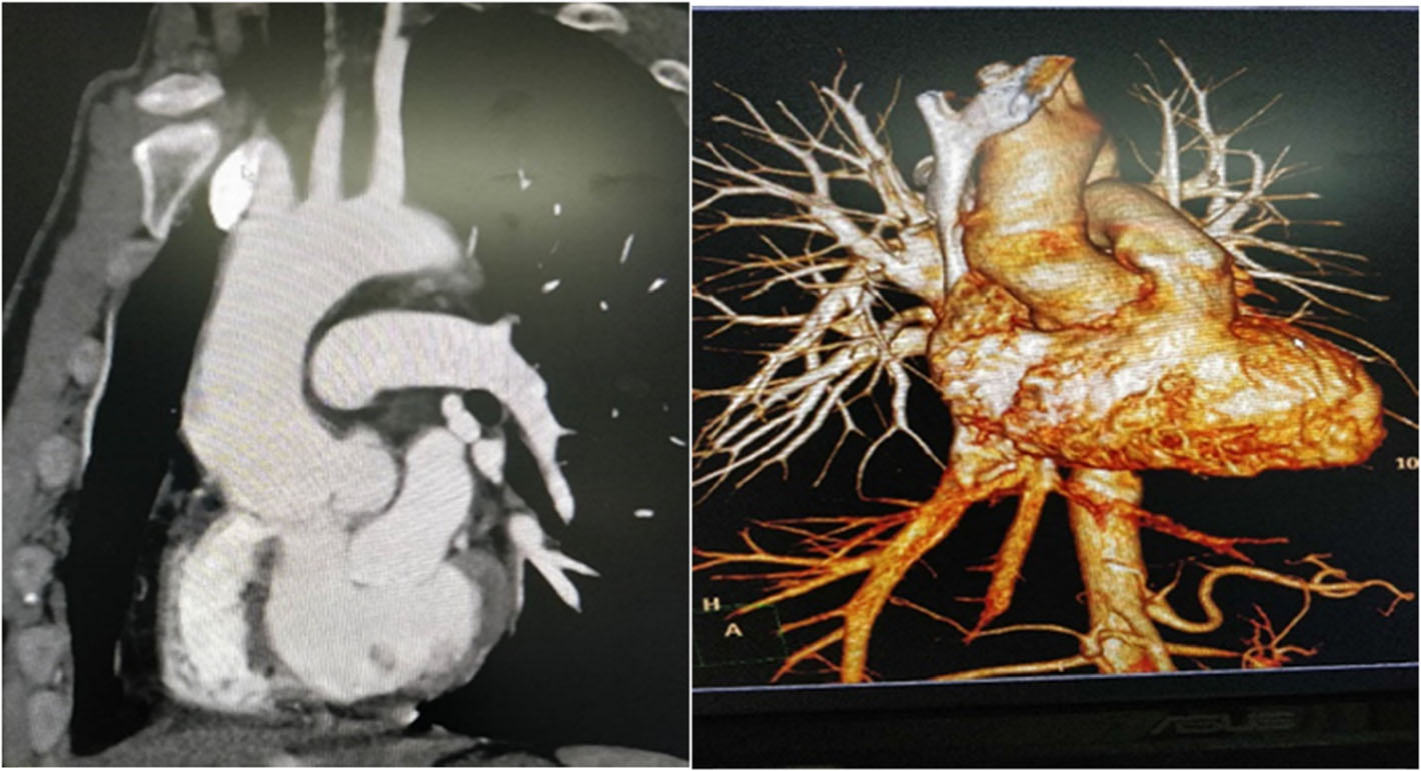
CTA scan indicated the aortic sinus is cystic outwards

A-39-year old Chinese man was admitted to our hospital for repeated oral ulcers and headaches for 8 years, chest pain for 7 months. He had no diabetes, no relevant medical family history, and no external genital ulcer. The laboratory test results: C-reactive protein of 32.3 mg/L (normal value:<5 mg/L), anti-nuclear antibody (ANA) was positive (normal value: negative), ESR of 55 mg/h (normal value: male: 0-15 ml/h, female: 0-20 ml/h). Transthoracic echocardiography (TTE) demonstrated: aortic sinus was 35 × 57 mm, ascending aorta diameter was 37 mm, at the junction of right and left coronary sinus there was a 12 × 14 mm cystic structure was formed outside from aortic wall, and a 40 × 23 mm cystic structure was formed at the junction of orifice of coronary sinus, as shown in Fig. 1. CTA scan indicated that the aortic sinus was outwards, the large cross-section area about 4.4 cm × 2.6 cm, as shown in Fig. 2. After admission to the hospital, he was treated with Glucocorticoid, Thalidomide, and Atorvastatin in the rheumatic immunology department until the inflammatory markers returned to a normal level, then he received modified Bentall surgery and continue to take medicine as pre-operation. After 8 months follow-up, the patient recovered well: TTE indicated artificial blood vessel has no apparent abnormalities and artificial heart valve is functioning well, no perivalvular leakage (PVL), eject fraction is 62%.

**Fig. 3:**
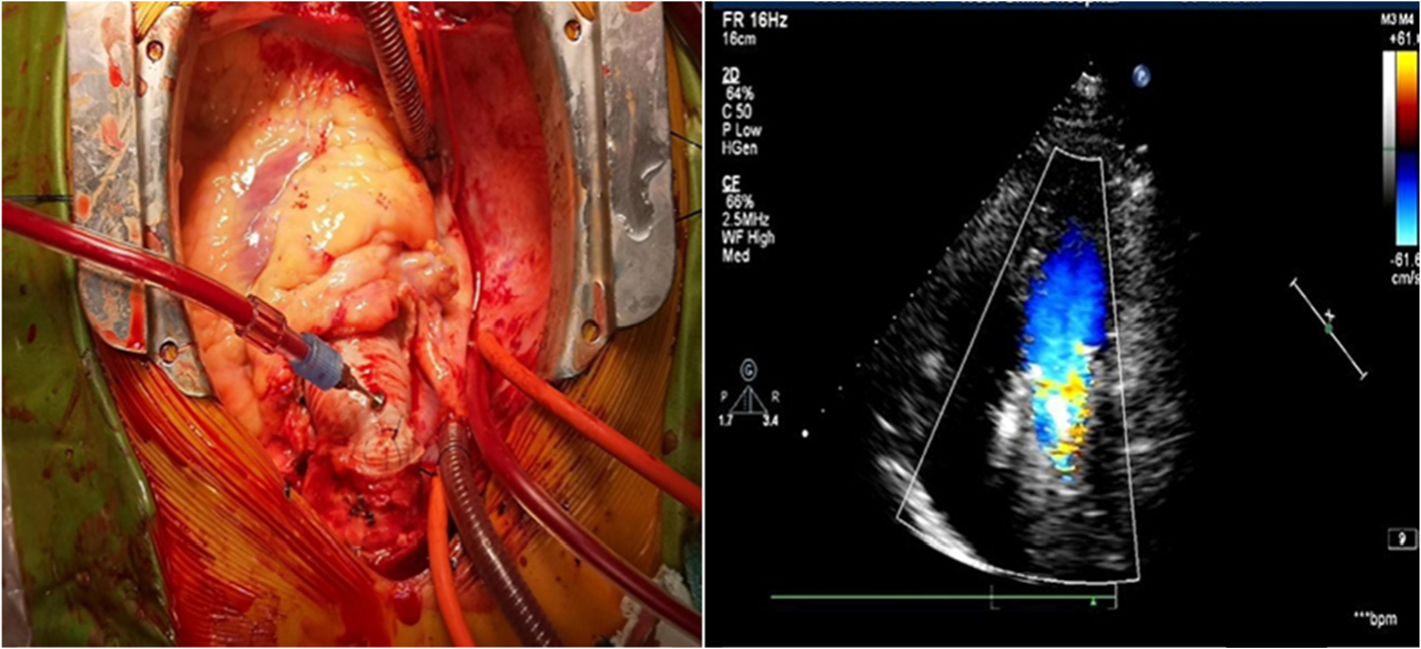
TEE indicated that the aortic valve regurgitation disappeared

Surgery process: median sternotomy and establish total cardiopulmonary bypass (CPB), myocardial protection with cold blood cardioplegia. Open the ascend aorta, cut the brachiocephalic artery, the native root including the annulus was excised, aortic root replacement with the modified Bentall technique was performed: The valved conduit procedure was a modified Bentall operation where the aortic mechanical valve prosthesis was sutured into the graft at 1 cm from the end of the graft with a continuous 3–0 polypropylene suture, forming a composite graft, which was directly sutured to the left ventricular outflow tract with a continuous 3–0 polypropylene suture other than to annulus, and then the composite graft was fixed by outside the aortic wall with a belt-like Teflon felt. The coronary buttons were anastomosed to the composite valve graft end-to-side with continuous suture used a 5–0 polypropylene suture without any tension, at last, the distal end of the conduit was anastomosed to the distal ascending aorta with continuous 3–0 polypropylene sutures. The CPB and aortic cross-clamp times were 117 min and 60 min respectively. During this procedure no difficult bleeding encountered. There was no obvious abnormality in the function of artificial mechanical valves, and artificial ascending aortic blood flow was smooth, TEE suggested the aortic valve mechanical valve worked well, as shown in Fig. 3. Postoperative pathological indicated that the inner layer of the arterial wall was uneven, with partial fibrous hyperplasia, focal mucus degeneration, and a few lymphocytes infiltration. Immunohistochemical: smooth muscle cells were positive, CD3 + lymphocyte infiltration. Web dyeing: elastic fibers were positive, which suggested aseptic inflammatory changes in the aorta.

## Discussion

BD is a chronic systemic inflammatory disease of unknown etiology. Clinical manifestations mainly include recurrent oral and genital diseases, skin ulcers, and so on [1]. The disease is distributed worldwide, but it presents an important regional difference in incidence rate. The Mediterranean, Middle East, and the Far East have the highest incidence rate, so it is also called “Silk Road disease.” [2] The incidence rate in China is about 0.0001% ~ 0.0004%, mainly found in men between the ages of 20 and 40 [3, 4]. The basic pathological manifestations are: non-specific systemic vasculitis, including arteries and veins of any diameter, significant neutrophils and monocytes to infiltration, swelling of endothelial cells, and fibrin necrosis.

BD involves large arteries can lead to thromboses, occlusions and aneurysms [5], aneurysms rupture leads to death in most cases [6, 7], the main reasons including blood vessel inflammation and the nutrient vessels of the artery are damaged, which jointly damage to the arterial wall. BD also causes lesions in the aortic valves, results to aortic regurgitation because of vasculitis, which can be seen in5–19% patients with BD [8]. BD involves coronary artery is very rare, only a few cases reported [9–11]. Saadoun. D reported a seven-year follow-up study of 817 patients found that male and arterial lesions were associated with increased mortality [12].

The treatment of BD including both drugs and surgery. Corticosteroid and immunosuppressive drugs have been widely used for BD, and they are suggested before and after surgery because it will decrease the incidence of recurrent inflammation [13]. Anti-tumor necrosis factor agents, and interferon-α are also used for the treatment of BD recently [14]. Surgical treatment of aortic root involvement needs taking many factors into consideration, including surgical timing, procedure and materials, and perioperative management. The original Bentall procedure is used to repair aortic dissection which affecting the aortic root and valve, but it was related to the risk of false aneurysm formation and coronary separation, even re-operation [15, 16]. Aortic valve replacement/repair (AVR) in BD requires more considerations for postoperative complications such as valve detachment and pseudoaneurysm formation, which are fatal sometimes. Major bleeding, PVL, detachment of the prosthetic valve, and Pseudoaneurysm formation after Bentall procedure or AVR are happened inevitably in most cases mainly because recurrent inflammation increase fragility of the aortic wall and aortic annular tissue, as well as reduction in prostacyclin production, besides, the nutrient vessels of the artery are also evolved by inflammation, both of which result to the injury of the arterial wall, so it is difficult to repair the aortic for pseudoaneurysm or hemorrhage at the needle eyes may form on the vessel wall after needle puncture.

To avoid valve detachment, many various technical modifications have been devised, including using a valved conduit or homograft, fix the prosthetic valve at the aortic annulus via buttress sutures, suture a belt-like Teflon felt on the lateral side to reinforce the valve, but both of those techniques have a common character that the valve or homograft is sutured directly on the aortic annulus or aortic wall, patients underwent those modifications also have an extremely poor prognosis. Ando, et al. [17] reported valve detachment happened after AVR in 36% of those with BD, in 20% patients who underwent valved conduit procedure. Kouchoukos, et al. [18] first described button Bentall procedure to prevent pseudo-aneurysm formation, it was more difficult and time consuming but was related to higher survival rate [16]. Ando, et al. [17, 19] reported their MBP that the aortic valve prosthesis was sutured into the graft at 1–2 cm from the end of the graft via a continuous 3–0 polyester suture, so the composite graft was produced, then implanted into the aortic valve annulus use 2–0 polyester buttressed mattress sutures, and place a circumferential belt-like felt outside the aortic wall. They used button technique to reconstruct the coronary arteries. No one of seven patients died during the follow-up 138 months.Okada [20] reported valve detachment happened in 1/8 patients who underwent this modified Bentall technique. Chen and colleges [21] devised a modified Bentall procedure that they inserted A 6-F Foley catheter into the lumen of each coronary artery, under this Foley catheter guidewire, both coronary buttons were constructed with a 0.5 to 0.8 cm diameter cuff of the aortic wall and were mobilized over a short length to facilitate reimplantation. Excised the root totally, a valved conduit directly sutured to the left ventricular outflow tract instead of the fragile annulus to prevent recurrent prosthetic valve detachment in BD patients. They completed posterior half first, and then the coronary buttons were reimplanted to the valved conduit, without any tension during the whole process. at last, the distal end of the valved conduit was anastomosed to the distal ascending aorta. They used their Bentall procedure in 5 BD patients with postoperative aortic prosthetic valve detachment and achieved satisfactory short-term and mid-term results. MBP have many advantages than the normal Bentall procedure, such as MBP can reduce major bleeding risk, prevent coronary arteries from kinking, decrease tension on button coronary anastomosis, avoid false aneurysms development, reduce operative time, and lower morbidity and mortality rate.

Nowadays, although many new surgical techniques have been used, it seems that valve detachment and pseudoaneurysm formation are not avoidable. Fortunately, there are more and more reported cases indicate it is beneficial for BD patient to control inflammatory activity by corticosteroids and immunosuppressive drugs, before and after the procedure, CRP is usually recommended as a standard biomarker to evaluate rheumatic activity.

## Conclusion

we report a patient of BD combined with severe aortic valve regurgitation and two giant pseudoaneurysms of the aortic sinus. Preoperative used of corticosteroids and immunosuppressants until the inflammatory markers were diminished and then the modified Bentall procedure was successfully performed to replace the aortic root, used corticosteroids and immunosuppressants after surgery in order to decrease the recurrent incidence of aneurysm, Hatemi G and Balcioglu O [22, 23] recommended medical treatment at least 2 years is effective, our patient recovered well after 8 months follow up.

## Data Availability

The dataset of this case report is available from the corresponding author on reasonable request.

## Abbreviations

AVR: Aortic valve replacement
BD: Behcet’s disease
CPB: Cardiopulmonary bypass
CT: Computed tomography
MBP: Modified Bentall procedure
PVL: Perivalvular leaks
TTE: Transthoracic echocardiography
